# Modified pulmonary artery banding: A novel strategy for balancing pulmonary blood flow with transposed great arteries

**DOI:** 10.1016/j.xjtc.2021.05.021

**Published:** 2021-06-02

**Authors:** Gananjay G. Salve, Andrew D. Cole, Ian A. Nicholson, David S. Winlaw, Richard B. Chard, Yishay Orr

**Affiliations:** a Heart Centre for Children, The Children's Hospital at Westmead, Westmead, New South Wales, Australia; b Faculty of Medicine and Health, The University of Sydney, Sydney, New South Wales, Australia; c Cincinnati Children's Hospital Medical Center, Cincinnati, Ohio

**Keywords:** transposition of the great arteries, arterial switch operation, modified pulmonary artery banding, biventricular repair, univentricular repair, ASO, arterial switch operation, BDGS, bidirectional Glenn shunt, BVR, biventricular repair, Cx, circumflex artery, d-TGA, dextro transposition of the great arteries, IQR, interquartile range, LPA, left pulmonary artery, l-TGA, levo transposition of the great arteries, mPAB, modified pulmonary artery banding, PA, pulmonary artery, PAB, pulmonary artery banding, PTFE, polytetrafluoroethylene, RPA, right pulmonary artery, RV, right ventricle/ventricular, UVR, univentricular repair, VSD, ventricular septal defect

## Abstract

**Objective:**

To study the outcomes of a novel modified pulmonary artery banding (mPAB) technique used for staged repair of a subset of patients with complex transposition physiology.

**Methods:**

A total of 13 patients who underwent mPAB during their staged repair (biventricular repair [BVR], n = 6) or palliation (1-1/2 repair, n = 1; univentricular repair [UVR], n = 6) from 2004 to 2020 were studied retrospectively. A restrictive interposition graft was used to reconstruct the main PA between the pulmonary root and the distal pulmonary confluence, functioning as a mPAB. Twelve of the 13 patients (92.3%) underwent a concurrent arterial switch operation (ASO), of which 6 were palliative ASOs for 1-1/2 repair (n = 1) or UVR (n = 5). Patient weight and cardiac anatomy determined the size of interposition graft.

**Results:**

The disease spectrum included dextro transposition of the great arteries (d-TGA) with multiple ventricular septal defects (VSDs) (n = 4), Taussig–Bing anomaly (n = 3), d-TGA with VSD and hypoplastic right ventricle (RV) (n = 3), double-inlet left ventricle with l-TGA (n = 2), and congenitally corrected TGA with double-outlet RV (n = 1). The Lecompte procedure was performed in 10 patients. Predischarge echocardiography revealed a band gradient of 61 mm Hg (interquartile range [IQR], 40-90 mm Hg) for BVR/1-1/2 ventricular repair (n = 7) and 49 mm Hg (IQR, 37-61 mm Hg) for UVR (n = 6). Survival was 100% at a median follow-up of 3.7 years (IQR, 2.6-4.0 years).

**Conclusions:**

The mPAB technique is effective and reproducible for staged BVR or UVR for patients with TGA. It effectively regulates pulmonary blood flow, may reduce neopulmonary root distortion, and eliminates complications associated with band migration in standard PAB.

## Graphical abstract

**Figure:**
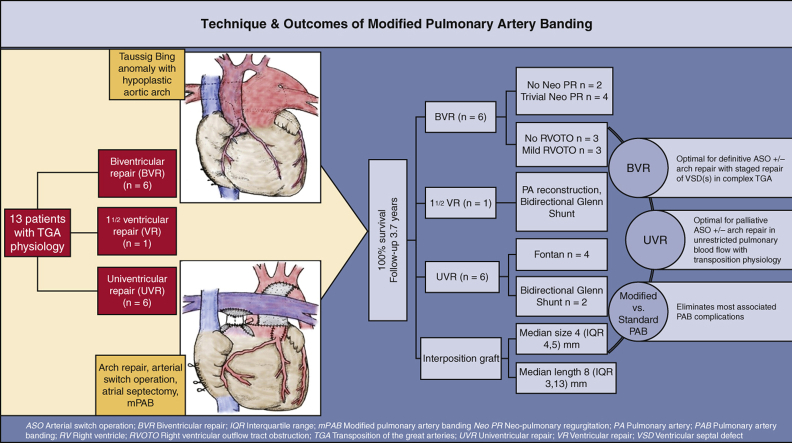

**Figure:**
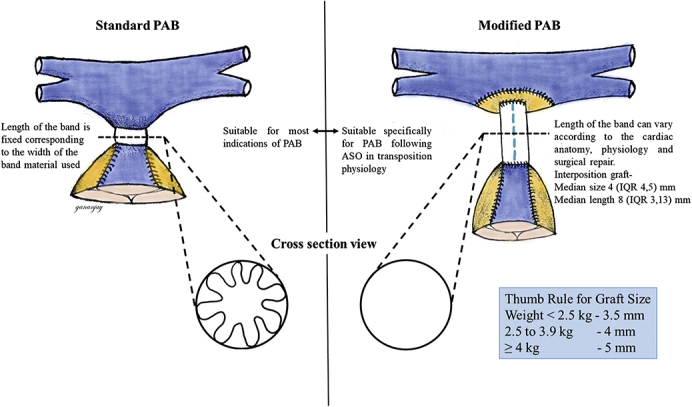
Sketch diagram depicting differences in standard and modified pulmonary artery banding.

See Commentaries on pages 121, 124, and 126.

**Central Message**

The novel technique of modified pulmonary artery banding (PAB) effectively restricts pulmonary blood flow with transposition physiology after an arterial switch operation while avoiding complications of standard PAB.

**Perspective**

Modified pulmonary artery banding (PAB) when performed in transposed great vessel physiology following a definitive or palliative arterial switch operation (ASO) functions better with both the diameter and the length of the interposition graft contributing to restriction of pulmonary blood flow. It eliminates the possibility of band migration associated with standard PAB and may reduce neopulmonary root distortion after ASO.

Pulmonary artery banding (PAB) is a palliative cardiac surgical technique used as an interim approach for the surgical management of a range of congenital heart defects associated with excessive pulmonary blood flow. Since its introduction,^1^ this technique has been widely used as an initial surgical intervention for infants born with cardiac defects characterized by left-to-right shunting and pulmonary overcirculation. Band tightness varies depending on the specific indication, either univentricular palliation or biventricular staged repairs.^2^ Over the last 2 decades, early definitive intracardiac repair has largely replaced palliation with PAB. This trend has evolved because many centers have demonstrated improved outcomes with primary corrective surgery as an initial intervention in neonates with congenital heart disease. Although the use of PAB has decreased significantly recently, it continues be an important therapeutic option in certain subsets of patients with congenital heart disease.

One such indication is the presence of multiple ventricular septal defects (VSDs) with dextro transposition of the great arteries (d-TGA).^3^^,^^4^ Traditional PAB is challenging with concurrent arterial switch operation (ASO) due to placement of the band on the reconstructed neopulmonary root and associated difficulty in achieving stable band position and adequate band tightness. In single ventricle anatomy with d-TGA, right ventricular (RV) hypoplasia, and VSD, transposition streaming often limits the ability to achieve sufficient band tightness because of consequent cyanosis. A palliative ASO^5^ may be useful in this situation to enable effective restriction of pulmonary blood flow without unacceptable cyanosis. Similarly, univentricular levo (l)-TGA with systemic outflow tract obstruction requiring aortic arch repair and restriction of pulmonary blood flow also can be managed with palliative ASO and PAB. Consequently, we developed a novel technique of modified PAB (mPAB)^6^ to deal with these challenging anatomic substrates requiring restriction of pulmonary blood flow concurrent with definitive or palliative ASO.

In this study, we evaluated the indications, technical aspects and outcomes of mPAB since its inception at our institution for patients with transposition physiology who required either univentricular palliation or staged biventricular repair (BVR).

## Methods

### Patients

Between August 2004 and June 2020, 13 patients with transposition anatomy combined with VSD(s) or single ventricle physiology producing unrestrictive pulmonary blood flow underwent mPAB to restrict pulmonary overcirculation at the Children's Hospital at Westmead. Patients were retrospectively identified from institutional cardiac surgical databases, and the study was approved by the Sydney Children's Hospitals Network Human Research Ethics Committee (reference no. 2020/ETH01912). The need for informed consent was waived.

Neonates were stabilized in the neonatal intensive care unit, and mPAB was performed, with concurrent definitive or palliative ASO in all but 1 case. Interposition graft length was measured by reviewing postoperative imaging (2-dimensional echocardiography and/or computed tomography scan of the chest).

### Surgical Technique

**Figure 1:**
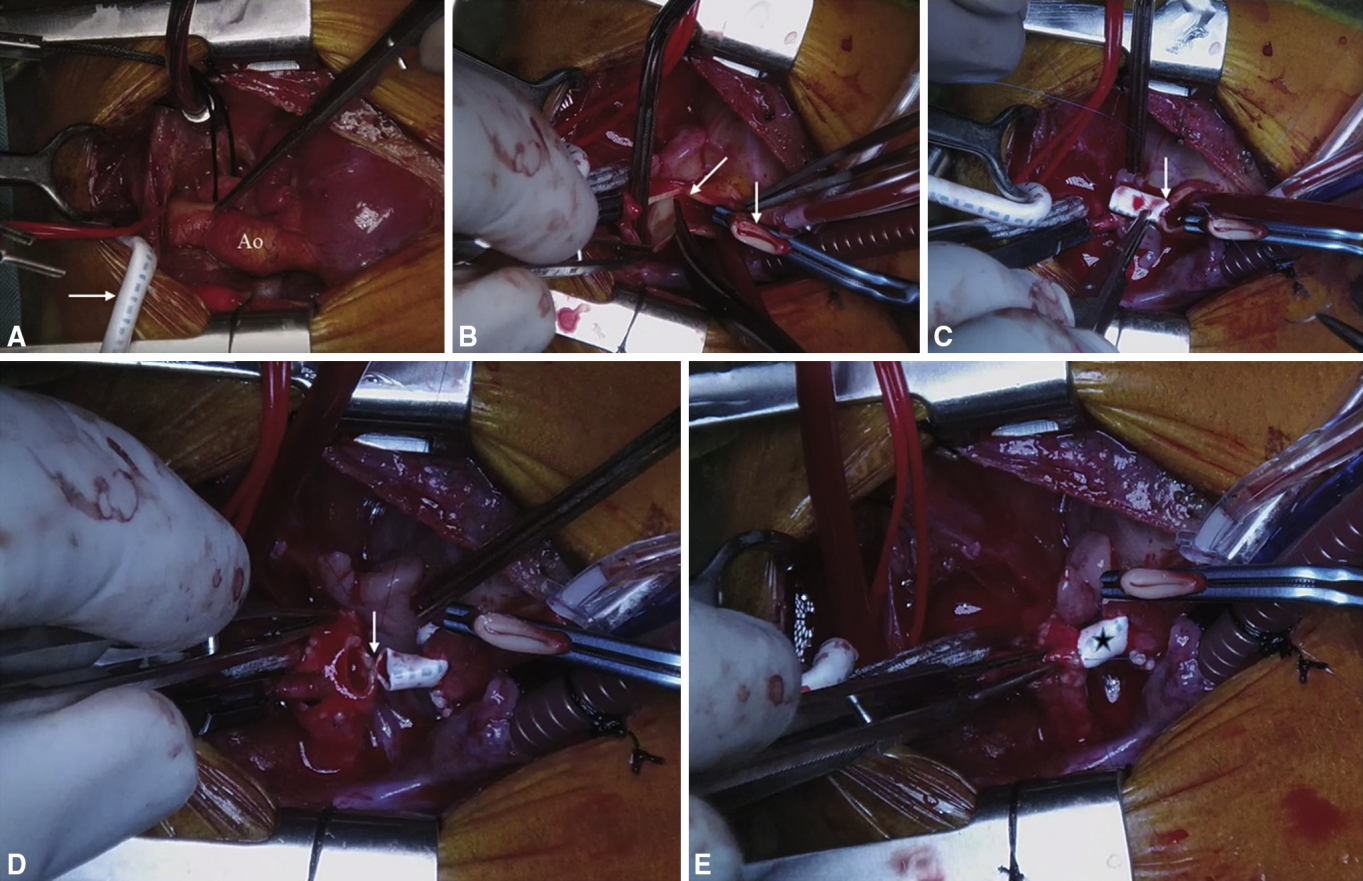
Intraoperative photos of modified pulmonary artery banding (mPAB). (A) A heart with a Taussig–Bing anomaly with the ascending aorta (Ao) anterior and to the right of the pulmonary artery (PA), along with aortic arch hypoplasia for which a polytetrafluroethylene (PTFE) graft (*transverse arrow*) has been anastomosed to the innominate artery. The silk suture is looping the patent ductus arteriosus. (B) A divided ascending aorta with a clamped aortic root (*vertical arrow*) and division of the PA at the confluence (*oblique arrow*). (C) The PTFE graft being anastomosed to the pulmonary root (*vertical arrow*). (D) The PTFE graft being anastomosed to a punch hole in the bovine patch at the pulmonary confluence (*vertical arrow*). (E) The completed interposition graft as the mPAB (*star*).

**Figure 2:**
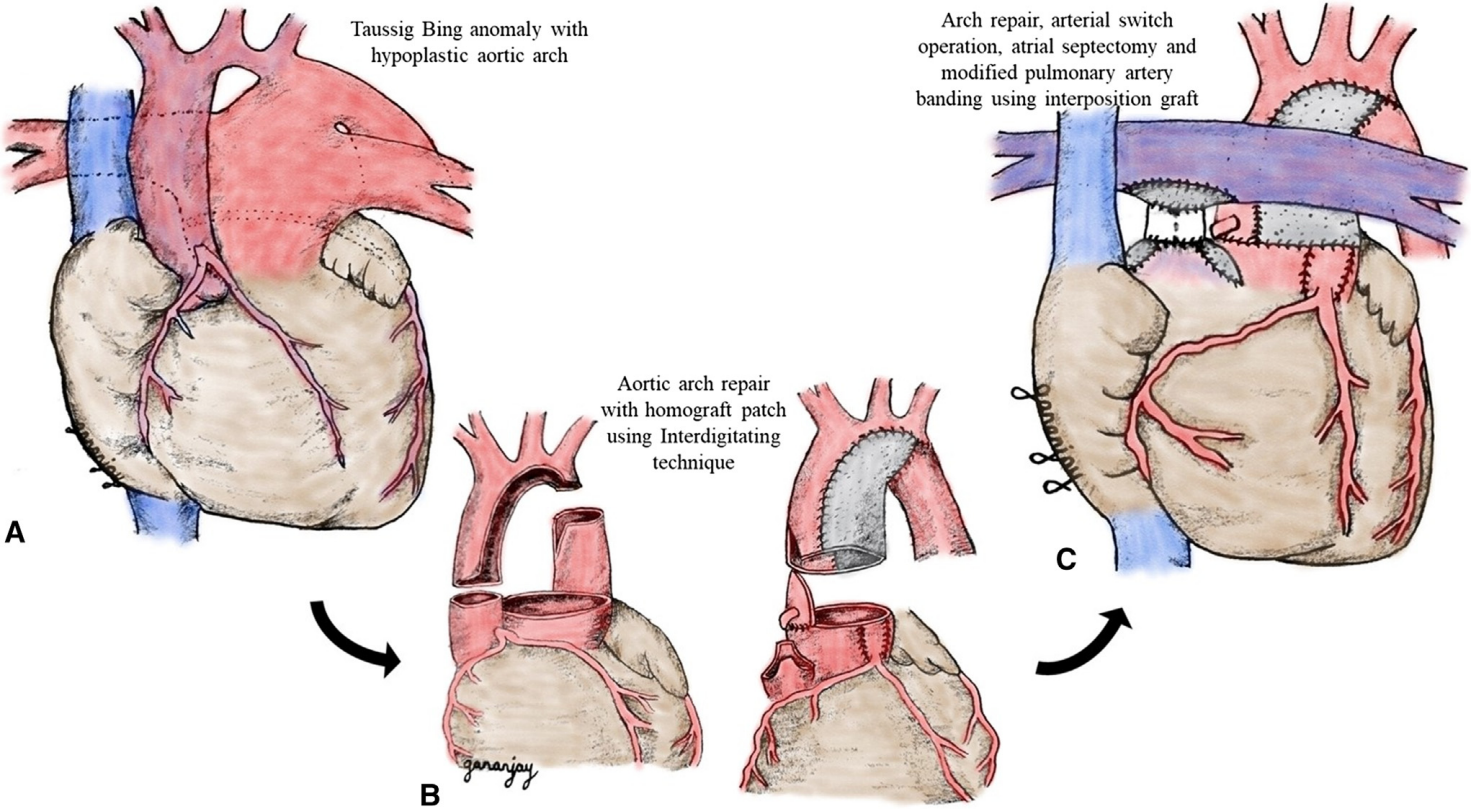
(A) Sketch showing a Taussig–Bing anomaly with hypoplastic aortic arch. (B) Aortic arch augmentation using the interdigitating technique with a homograft patch. (C) Aortic arch repair, arterial switch operation, atrial septectomy, and modified pulmonary artery banding using an interposition graft.

Through a median sternotomy, standard cardiopulmonary bypass was established under systemic heparinization, and coronary transfer with aortic reconstruction was performed following routine steps. For concomitant aortic arch repair, innominate artery cannulation was performed with a polytetrafluoroethylene (PTFE) side graft (Figure 1, *A*), and the arch was then reconstructed using the interdigitating technique^7^ with anastomoses between the aortic arch, distal aorta, and an anteroinferior augmentation patch of a pulmonary homograft (Figure 2, *A* and *B*).

The Lecompte maneuver was performed selectively, the ascending aorta was anastomosed to the neoaortic root, and the defects in the facing sinuses of the neopulmonary root were reconstructed with autologous pericardial patches. Pulmonary arterial continuity was then reestablished using the mPAB technique (Figure 2, *C*).

### mPAB With Interposition Graft

A suitably sized PTFE tube graft was anastomosed end-to-end to the reconstructed neopulmonary root, thereby gathering the sinotubular junction of the neopulmonary root onto the much smaller PTFE graft. This required a significant amount of nearing and faring of sutures to compensate for the size mismatch (Figure 1, *B* and *C*). In 1 patient, the neopulmonary root was too large for this technique, so a homograft patch was first sutured to close it. Then a central fenestration of 5 mm was created into the patch, and the PTFE graft was then anastomosed to this fenestration. In another patient (as demonstrated in Video 1), the proximal end of the interposition graft was splayed open with 3 equidistant cuts and then sutured to the pulmonary root to try and match the suturing distance on each end. The PTFE graft was then trimmed to a length suitable to construct a tension-free connection to the distal pulmonary artery (PA). A patch of pulmonary homograft, autologous pericardium, or bovine pericardium with a central fenestration was used to close the opening at the pulmonary confluence. The distal end of the PTFE tube graft was then anastomosed to the fenestration in the patch to reconnect the neopulmonary root to the distal PA (Figure 1, *D* and *E*). The diameter and length of the interposition graft were tailored to the each patient's weight and underlying anatomy.

**Video 1:**
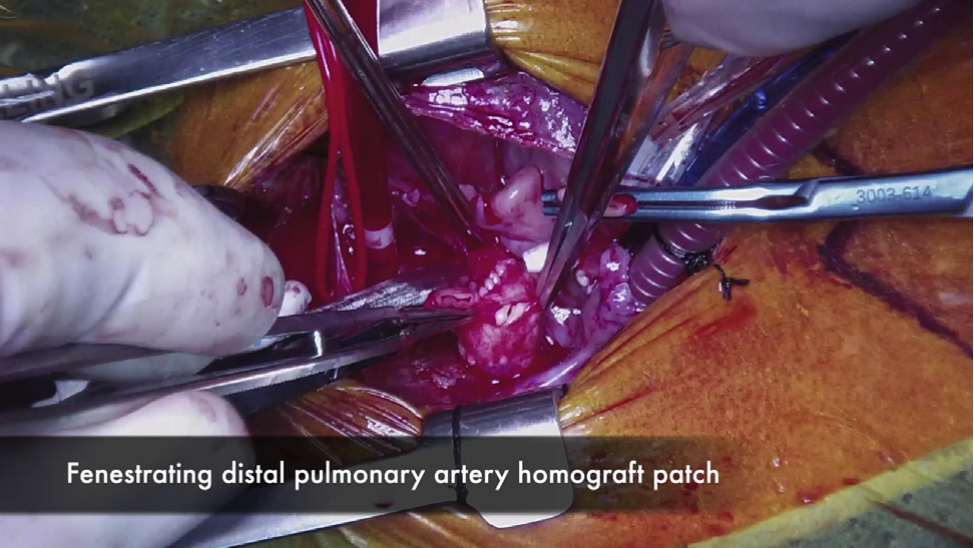
Operative video demonstrating the technique of modified pulmonary artery banding (mPAB) in a neonate with d-transposition of the great arteries necessitating mPAB for straddling the mitral valve chordae across a large ventricular septal defect. Video available at: https://www.jtcvs.org/article/S2666-2507(21)00388-6/fulltext.

### mPAB in Delayed Repairs

There were two patients in whom mPAB was performed following delayed presentation of single ventricle physiology. The first patient had d-TGA, a hypoplastic RV, and a large inlet VSD with a straddling atrioventricular valve not amenable to biventricular repair (BVR). The patient had been initially palliated at another institution with traditional PAB via left thoracotomy but had persistently elevated pulmonary vascular resistance, contraindicating single ventricle palliation with a venous shunt. At 1 year of age, he underwent a palliative ASO with mPAB, atrial septectomy, and open lung biopsy at our institution to manage unfavorable transposition streaming and better restrict pulmonary blood flow to remodel the pulmonary vascular bed. He ultimately proceeded to successful staged completion of total cavopulmonary connection.

The second patient had a double-inlet left ventricle with l-TGA. He presented late at age 6 years with pulmonary hypertension but dynamic pulmonary vascular resistance and so underwent traditional PAB as an initial palliation, followed by bidirectional Glenn shunt (BDGS) with mPAB to further restrict antegrade pulmonary blood flow as stage II palliation and then extracardiac Fontan completion as stage III palliation.

### Debanding for BVR

Redo sternotomy and closure of VSD(s) was done in standard fashion using cardiopulmonary bypass. The interposition PTFE tube graft and previously placed patch at the pulmonary confluence were then completely excised. Vertical incisions were made into each pulmonary valve sinus across the restricted sinotubular junction. Each of these sinuses was then augmented with a patch of autologous/bovine pericardium sutured in place to reconstruct a normal-sized pulmonary root, which was then anastomosed to the distal pulmonary confluence. After weaning from cardiopulmonary bypass, direct RV and PA pressures were measured to define any residual gradients.

### Debanding for 1-1/2 Ventricular Repair

This patient had d-TGA, moderate RV hypoplasia, a large muscular VSD, aortic coarctation, and arch hypoplasia and had undergone initial ASO with mPAB and concomitant aortic arch repair. At stage II, the VSD and the atrial septal defect were closed, and the pulmonary root was reconstructed as described above. A right-sided BDGS was then constructed in a standard fashion along with bovine pericardial patch augmentation of a hypoplastic right PA (RPA).

### Debanding and Interruption of Antegrade Pulmonary Blood Flow for Univentricular Palliation

Debanding and interruption of antegrade pulmonary blood flow was undertaken at either stage II (BDGS) or stage III (completion Fontan) palliation. The debanding was always carried out under aortic cross-clamping because of the univentricular anatomy. The interposition graft was removed from the distal anastomosis, and the opening in the pulmonary confluence was closed either directly or using a bovine pericardial patch. The proximal end of the graft was removed, the pulmonary valve cusps were excised, and the pulmonary root was closed directly.

### Statistical Analysis

Data are presented as median and interquartile range (IQR) unless stated otherwise.

## Results

### Descriptive Analyses

The spectrum of morphologic diagnoses included d-TGA and multiple VSDs (n = 4); Taussig–Bing anomaly (n = 3); d-TGA, VSD, and hypoplastic RV (n = 3); double-inlet left ventricle with l-TGA (n = 2); and congenitally corrected TGA with double-outlet RV (n = 1) (Table 1). A total of 6 patients had a hypoplastic RV, of whom 5 patients underwent univentricular repair (UVR) and 1 patient underwent 1-1/2 ventricular repair. The aortic arch was normal in 6 patients and hypoplastic in 7 patients (53.8%). The great artery relationship was anteroposterior in 10 patients (77%) and side-by-side in the remaining 3 (23%). The coronary artery pattern was 1 left anterior descending artery (L) circumflex artery (Cx); 2 right coronary artery (R) in 9 patients, 1L;2RCx in 2 patients, RLCx from a sinus in 1 patient, and a single coronary ostium in 1 patient.

**Table 1:** All morphological diagnoses

Patient	Primary diagnosis	Great artery relationship	VSD type	Aortic arch	Coronary pattern	Additional diagnosis
A	Taussig–Bing anomaly	SS	Multiple	Hypoplastic arch	1LCx;2R	Bilateral SVC
B	Taussig–Bing anomaly	AP	Multiple	Normal	1LCx;2R	-
C	d-TGA/multiple VSDs	AP	Multiple muscular	Normal	1LCx;2R	-
D	d-TGA/multiple VSDs	AP	Multiple muscular	Normal	1L;2RCx	-
E	d-TGA/multiple VSDs	SS	Multiple muscular	Normal	Single coronary ostium	-
F	d-TGA/multiple VSDs	AP	Large subpulmonic, posterior muscular	Hypoplastic arch	1LCx;2R	Mild dysplastic pulmonary valve, mild pulmonary regurgitation
G	d-TGA/VSD/hypoplastic RV	AP	Large muscular	Coarctation of aorta	1LCx;2R	Mild dysplastic pulmonary valve
H	d-TGA/VSD/hypoplastic RV/tricuspid atresia	AP	Small muscular	Hypoplastic arch	1LCx;2R	Ventricular inversion
I	ccTGA/DORV/VSD	AP	Large Inlet	Coarctation of aorta	2RCxL	VSD not amenable to BVR
J	Taussig–Bing anomaly/hypoplastic RV	AP	Large outlet	Hypoplastic arch	1LCx;2R	-
K	d-TGA/VSD/hypoplastic RV/previous standard PAB	AP	Large inlet	Normal	1L;2RCx	Straddling AV valve
L	DILV/d-TGA/hypoplastic RV	SS	Large outlet	Coarctation of aorta	1LCx;2R	-
M	DILV/l-TGA/PHTN/previous standard PAB	AP	Large outlet	Normal	1LCx;2R	Late presentation with PHTN

*VSD*, Ventricular septal defect; *SS*, side-by-side; *L*, left anterior descending coronary artery; *Cx*, circumflex coronary artery; *R*, right coronary artery; *SVC*, superior vena cava; *AP*, anteroposterior; *d-TGA*, dextro transposition of the great arteries; *RV*, right ventricle; *ccTGA*, congenitally corrected transposition of the great arteries; *DORV*, double-outlet right ventricle; *BVR*, biventricular repair; *PAB*, pulmonary artery banding; *AV*, atrioventricular; *DILV*, double-inlet left ventricle; *l-TGA*, levo transposition of the great arteries; *PHTN*, pulmonary hypertension.

Preoperative patient demographics are summarized in Table 2. Three patients underwent pre-ASO procedures, including 1 device closure for multiple VSDs and traditional PAB in 2 patients, 1 via thoracotomy at another institution, and 1 via sternotomy as initial palliation at our institution. The operative procedure performed at the time of mPAB and the details of interposition graft used are summarized in Table 3. The Lecompte procedure was performed in 10 patients (77%). Reasons for not performing a Lecompte procedure were age outside the neonatal period (n = 2) or an unusual branch PA configuration limiting pulmonary arterial mobility (n = 1).

**Table 2:** Patient characteristics

Characteristic	Value
**Preoperative characteristic**	
Antenatal diagnosis, n/N (%)	7/13 (54)
Males:females, n	9:4
Birth weight, kg, median (IQR)	3.3 (2.5-3.9)
Gestational age, wk, median (IQR)	39 (38-40)
Prostaglandin infusion, n/N (%)∗	9/11 (82)
Balloon atrial septostomy, n/N (%)∗	3/11 (27)
Pre-ASO ventilation, n/N (%)†	4/12 (33)
Weight at ASO, kg, median (IQR)†	3.3 (2.7-4.0)
Age at ASO, d, median (IQR)†	15 (6-21)
**Intraoperative characteristics at operation for mPAB**	
Cardiopulmonary bypass time, min, median (IQR)	250 (240-262)
Aortic cross-clamp time, min, median (IQR)	122 (115-129)
Delayed sternal closure, n/N (%)	9/13 (69)
**Postoperative characteristics following mPAB**	
Ventilation time, h, median (IQR)	118 (102-339)
Intensive care unit stay, h, median (IQR)	142 (114-373)
Hospital stay, d, median (IQR)	20 (19-42)

*IQR*, Interquartile range; *ASO*, arterial switch operation; *mPAB*, modified pulmonary artery banding.

∗ 11 out of 13 patients in the cohort were neonates at the time of the mPAB procedure.

† 12 out of 13 patients in the cohort underwent either ASO or palliative ASO.

**Table 3:** Operative procedure and interposition graft details

Patient	Primary procedure	Lecompte procedure	Body surface area, m^2^	Weight, kg	Age at primary procedure, d	Neo PA annulus size, mm	*z*- score	Graft size, mm	Graft length, mm	Graft diameter to weight ratio	Graft length to weight ratio	Predischarge band gradient, mm Hg
A	ASO, Arch repair, mPAB, atrial septectomy	Yes	0.17	2.30	3	6.7	−0.46	4	1.8	1.74	0.8	50
B	ASO, mPAB	Yes	0.20	3.03	46	7.8	0.05	5	3.0	1.65	1	40
C	ASO, partial VSDs closure (directly), ASD closure, mPAB	Yes	0.22	3.80	6	7.1	−1.03	4	7.1	1.05	1.9	61
D	ASO, mPAB	Yes	0.18	2.60	15	6.5	−0.87	3.5	1.4	1.35	0.5	90
E	ASO, partial VSDs closure (directly), ASD closure, mPAB	Yes	0.25	4.40	37	6.3	−2.14	5	15.0	1.13	3.4	74
F	ASO, Arch repair, mPAB	Yes	0.21	3.30	7	6.7	−1.12	4	3.0	1.21	0.9	33
G	Palliative ASO, Arch repair, mPAB	Yes	0.19	2.75	4	9.1	1.24	4	8.0	1.45	2.9	92
H	Palliative ASO, Arch repair, mPAB	Yes	0.18	3.15	3	6.0	−1.76	4	12.4	1.27	4	64
I	Palliative ASO, Arch repair, Atrial septectomy, mPAB, VSD left open	Yes	0.23	4.14	7	5.1	−3.39	5	12.9	1.21	3.1	37
J	Palliative ASO, Arch repair, mPAB	No	0.20	3.49	2	6.3	−1.64	5	27.7	1.43	8	37
K	Palliative ASO, mPAB, atrial septectomy, lung biopsy	No	0.57	7.60	377	10	−0.44	6	26.3	0.79	3.5	60
L	Palliative ASO, Arch repair, mPAB	Yes	0.17	2.20	9	4.4	−3.16	3.5	7.6	1.59	3.5	43
M	BDGS, mPAB, atrial septectomy, 8-mm graft restricted to 5-mm diameter	No	0.86	21.80	2771	18.9	0.84	8	21.0	0.37	1	55

*PA*, Pulmonary artery; *ASO*, arterial switch operation; *mPAB*, modified pulmonary artery banding; *VSD*, ventricular septal defect; *ASD*, atrial septal defect; *BDGS*, bidirectional Glenn shunt.

The median size of the interposition graft used was 4.0 mm (IQR, 4-5 mm), and the median length was 8.0 mm (IQR, 3-13 mm). Indexing interposition graft diameter to patient weight revealed a median ratio of 1.35 mm/kg (IQR, 1.13-1.65 mm/kg) for patients undergoing BVR/1-1/2 ventricular repair (n = 7) and 1.35 mm/kg (IQR, 1.22-1.55 mm/kg) for those undergoing UVR (n = 4). The median length of the grafts used for BVR/1-1/2 ventricular repair (n = 7) was 3 mm (IQR, 2-8 mm), whereas that for UVR (n = 4) was 13 mm (IQR, 9-24 mm). Indexing graft length to patient weight at the time of surgery for those undergoing neonatal procedures revealed a median ratio of 1 mm/kg (IQR, 1-3 mm/kg) for BVR/1-1/2 ventricular repair (n = 7) and 4 mm/kg (IQR, 3-7 mm/kg) for UVR (n = 4). The median *z*-score of neopulmonary annulus size for BVR/1-1/2 ventricular repair (n = 7) was −1.03 (IQR −1.24 to 0.46), and that for UVR (n = 4) was −2.46 (IQR, −3.33 to −1.67). Patients K and M were excluded from these analyses because they were older and presented to our institution later than the other patients in the study cohort. Intraoperative and postoperative demographics are detailed in Table 2.

The most common postoperative complications seen were low cardiac output state (n = 10), renal dysfunction requiring peritoneal dialysis (n = 4), and cardiac arrhythmias (n = 4) (Table 4). Predischarge echocardiography confirmed a median band gradient of 61 mm Hg (IQR, 40-90 mm Hg) for patients planned for BVR/1-1/2 ventricular repair (n = 7) and 49 mm Hg (IQR, 37-61 mm Hg) for those planned for UVR (n = 6).

**Table 4:** Postoperative complications

Complication	Frequency
Low cardiac output state	10
Renal dysfunction	4
Cardiac arrhythmias	4
Bleeding requiring reexploration	3
Chylothorax	3
Extra corporeal membrane oxygenation	2
Reexploration for mediastinal washout	1
Cardiac arrest	1
Wound infection	1

### Interim Catheter Procedures and Reinterventions

All patients had planned reinterventions, and total reinterventions (first, second, and third reinterventions) are summarized in Table 5. The median interval between the primary procedure and the first reintervention was 4.9 months (IQR, 2.4-9.9 months). For BVR, all 6 patients underwent planned PA debanding with excision of the interposition graft and reconstruction of the main PA at stage II. Four patients underwent concomitant closure of associated VSD(s), either single (n = 2) or multiple (n = 2). Three patients required concomitant branch PA plasty (2 left PA [LPA] and 1 RPA).

**Table 5:** Summary of reinterventions

Patient	Unplanned procedure at first reintervention	First planned reintervention					Second reintervention		Third reintervention	
		Time from primary procedure, mo	Procedure	Age, mo	Weight, kg	BSA	Procedure	Age, mo	Procedure	Age, mo
A	LPA plasty	4.9	PA debanding, VSD closure, and ASD closure	5	4.9	0.27	-	-	-	-
B	-	13.5	PA debanding and closure of large outlet VSD, mid-muscular VSD, and apical muscular VSD	15	8.2	0.41	-	-	-	-
C	MAPCA coiling in catheterization laboratory	1.8	PA debanding	2	5.01	0.26	B/L branch PA banding (unplanned)	13	Device closure of muscular VSD followed by B/L branch PA plasty (unplanned)	25
D	-	9.5	PA debanding and closure of mid-muscular VSD	10	7.12	0.35	-	-	-	-
E	RPA plasty, ECMO initiation	16.7	PA debanding	18	10.65	0.47	Device closure of large mid-muscular VSD and ECMO weaning (unplanned)	18	-	-
F	LPA plasty	4.8	PA debanding and closure of 2 VSDs	5	5.88	0.31	-	-	-	-
G	RPA plasty	7.9	PA debanding, VSD closure, ASD closure, and BDGS	8	7.14	0.36	-	-	-	-
H	Augmentation of LPA	2.4	BDGS	2.5	4.82	0.26	LPA stenting (unplanned)	4	Fenestrated Fontan procedure (planned)	44
I	Weaning of ECMO	0.06	PA band tightening	0.3	4.14	0.23	BDGS, pulmonary valvectomy, PA interruption (planned)	8	Fontan procedure with LPA stenting (planned)	42
J	-	9.9	BDGS, interposition graft left in situ	10	8.55	0.38	-	-	-	-
K	-	7.4	BDGS, removal of graft, and interruption of PA	20	9.6	0.46	Completion of Fontan procedure (planned)	38	-	-
L	RPA plasty	3.7	BDGS	4	5.4	0.28	-	-	-	-
M	-	35	Fontan procedure, removal of graft, and pulmonary valvectomy	127	26.8	1.01	Subaortic myectomy, anterior enlargement of VSD (unplanned)	191	-	-

*BSA*, Body surface area; *LPA*, left pulmonary artery; *PA*, pulmonary artery; *VSD*, ventricular septal defect; *ASD*, atrial septal defect; *MAPCA*, major aortopulmonary collateral artery; *B/L*, bilateral; *RPA*, right pulmonary artery; *ECMO*, extracorporeal membrane oxygenation; *BDGS*, bidirectional Glenn shunt.

The patient who underwent 1-1/2 ventricle repair (patient G) had a straightforward stage II procedure involving PA debanding and reconstruction, VSD closure, atrial septal defect closure, RPA augmentation, and BDGS anastomosis. Of those who underwent univentricular palliation (n = 6), the first reintervention involved BDGS (n = 4), branch PA augmentation (n = 1 LPA and n = 1 RPA), PA interruption (n = 2), and completion Fontan (n = 2). The second reintervention involved PA interruption followed by BDGS (n = 1), LPA stenting (n = 1), or completion Fontan (n = 1). One patient underwent completion Fontan along with LPA stenting as a hybrid procedure, and another patient underwent fenestrated Fontan as a third reintervention.

Patients C, E, and I had eventful recoveries following mPAB and required unplanned subsequent reinterventions (Table 5). While awaiting debanding, patient E developed significant RV dysfunction and still had a significant VSD. Preoperative cardiac computed tomography angiography was performed to image the branch PAs. Following extubation, after a computed tomography scan, a brief hypoxic cardiac arrest occurred owing to laryngospasm superimposed on underlying circulatory issues. After resuscitation, urgent redo sternotomy, and cardiopulmonary bypass, the main PA was reconstructed after excising the interposition graft, and the RPA origin was augmented. Device closure of the VSD was considered the optimal strategy, so the patient was transitioned to extracorporeal membrane oxygenation support and underwent device closure of a large mid-muscular VSD in the cardiac catheterization laboratory, followed by weaning from extracorporeal membrane oxygenation the next day.

Patient C had failed attempts at extubation following the debanding and PA reconstruction procedure. Cardiac catheterization study revealed an aortopulmonary collateral and a potentially significant muscular VSD. The collateral was occluded with a coil, and device closure of the VSD was attempted, which caused iatrogenic VSD enlargement. The patient then underwent bilateral branch PAB for stabilization as a second reintervention, followed by VSD device closure with branch PA reconstruction 1 year later.

Patient M required a post-Fontan subaortic myectomy with anterior enlargement of the VSD for progressive development of subaortic stenosis in the context of double-inlet left ventricle and l-TGA.

All patients were alive and well at the time of this report, at a median follow-up of 3.7 years (IQR, 2.6-4.0 years). The most recent echocardiography studies for all patients who underwent BVR (n = 6) showed no neopulmonary regurgitation in 2 patients and trivial to mild regurgitation in the other 4 patients. Three patients had no RV outflow tract obstruction, whereas the other 3 had RV outflow tract peak gradients of 12, 16, and 39 mm Hg.

## Discussion

The last decade has seen an increasing preference for early complete primary repair of complex congenital cardiac defects rather than staged repair with interim palliation. However, in some situations, anatomic complexity associated with a technically challenging repair favors a staged approach to provide physiologic stability and facilitate somatic growth to achieve a final successful definitive repair. In univentricular physiology with unrestricted pulmonary blood flow, transposition streaming limits the ability to achieve adequate restriction of pulmonary blood flow with traditional PAB owing to the consequent cyanosis. In these cases, it may be preferable to correct transposition streaming with a palliative ASO to enable adequate restriction of antegrade pulmonary blood flow.^5^ However, placing a PA band around a reconstructed neopulmonary root is not ideal, and, consequently, the technique of mPAB was developed at our institution.^6^ Over the last 16 years, the mPAB technique has been used selectively in 13 patients with transposition physiology at our institution, with a tailored approach to individual anatomy and physiology resulting in 100% survival.

We have found this technique to be particularly useful in the context of a palliative ASO to predictably regulate antegrade pulmonary blood flow rather than relying on native neopulmonary valvar or subvalvar obstruction, which can be dynamic and variable. mPAB is also useful for late-presenting univentricular anatomy with unrestricted pulmonary blood flow necessitating PAB as initial palliation. The main PA is very large and tense in this context, making it challenging to achieve adequate constriction and risking vascular injury with traditional PAB. There is probably less distortion of the neopulmonary valve and neopulmonary root with mPAB, because this technique avoids the infolding of the pulmonary arterial wall that can occur with standard external PAB. Complications inherent to traditional PAB, including distal band migration with compression of branch pulmonary arteries, proximal band migration with distortion of the neopulmonary valve, and coronary artery compression, are impossible with mPAB owing to the fixed placement of the interposition graft. The PTFE material used for banding (both standard and modified) inevitably incites scarring and adhesion formation; however, mPAB is advantageous, in that the band does not require removal from underlying structures but is simply excised.

In 1972, Trusler^2^ elucidated a formula to estimate appropriate band tightness based on band circumference but acknowledged the issues with transposition streaming and the need for a looser band in this context. Moreover, Trusler's rule was established in infants in heart failure with VSDs, not in neonates. It is widely used as a starting point for estimating PA band tightness; however, many other variables also should be considered, including estimating the pulmonary–systemic flow ratio^8^ and measuring distal PA pressure^9^ to ensure adequate band tightness. Our technique differs in that no band adjustment is required; a fixed diameter is selected based on patient weight and estimating at least a 50% luminal reduction in PA diameter in the region of the interposition graft. Graft sizes were usually 3.5 mm for patients weighing <2.5 kg, 4 mm for those at 2.5 to 3.9 kg, and 5 mm for those at ≥4 kg. The neopulmonary annulus size was also taken into consideration while sizing the interposition graft, especially for the UVRs, where there was significant discrepancy between the great vessel sizes. The length of the interposition graft was determined by the distance required to bridge the gap between the neopulmonary root and the pulmonary confluence, allowing a tension-free anastomosis regardless of whether the Lecompte maneuver was used.

The major difference in flow dynamics with mPAB versus traditional PAB is best explained by the Hagen–Poiseuille equation, Δ*p* = 8μLQ/πR^4^^,^^10^ which states that the pressure drop (Δ*p*) through a cylindrical tube is directly proportional to the length of the tube (L) and the volumetric flow rate of an incompressible fluid (Q) through the tube, but inversely proportional to the fourth power of radius of the tube (R). Consequently, the diameter (or radius) at the banded site is the most important determinant of band gradient regardless of the technique used. Intraluminal PAB also has been used in similar clinical situations^11^^,^^12^ however, despite the fixed diameter of flow restriction and capacity for catheter-based dilatation of the restriction to increase pulmonary blood flow, there is no length to the flow restriction, making the intraluminal band less titratable to individual patient anatomy and physiology. Importantly, the length of the band can be varied with the mPAB technique to further optimize flow restriction, whereas standard PAB will have a fixed short length defined by the width of the band material. As noted in Table 3, all 3 patients who did not undergo a Lecompte maneuver (patients J, K, and M) had longer interposition grafts, which permitted use of a larger-diameter graft to compensate for length-induced increases in the band gradient.

The goal for patients in our cohort with d- or l-TGA, single ventricle anatomy, and unrestricted pulmonary blood flow was to achieve unobstructed systemic outflow with aortic arch augmentation when required, correct transposition streaming and systemic outflow tract obstruction with palliative ASO, and then control the pulmonary blood flow with mPAB to enable staged Fontan palliation. In the 1 patient who did not undergo palliative ASO with mPAB (patient M), unfortunately, the pulmonary valve was sacrificed at Fontan completion rather than debanding and incorporating it into a Damus–Kaye–Stansel anastomosis. He later required subaortic resection. Importantly, mPAB facilitates long-term preservation of the pulmonary valve, particularly with downsizing of the interposition graft at interim stages to enable late incorporation in to a Damus–Kaye–Stansel anastomosis when required. All other patients had d-TGA with multiple VSDs, making complete primary repair challenging and the likelihood of residual defects high. We opted to use mPAB with ASO in this group to restrict left-to-right shunting and to allow spontaneous closure of small VSDs. Persistent large defects were then closed at a later stage with acceptable surgical/interventional risk with concurrent BDGS in the context of RV hypoplasia (n = 1).

After indexing the diameter of interposition graft to the weight of the patient, an identical ratio was obtained for BVR versus UVR; however, the graft was longer for univentricular conditions (Table 3). This can be explained in part by larger patient size and reduced use of the Lecompte maneuver in the univentricular group. The longer length of interposition graft in the univentricular patients was again evident after indexing the graft length to patient weight.

### Limitations

This study was retrospective in nature and subject to the limitations inherent to observational investigations. Data were derived from a single institution, which may limit the generalizability of our findings. The number of patients who underwent mPAB is small, as this technique is indicated for a very specific type of congenital cardiac disease physiology.

## Conclusions

**Figure 3:**
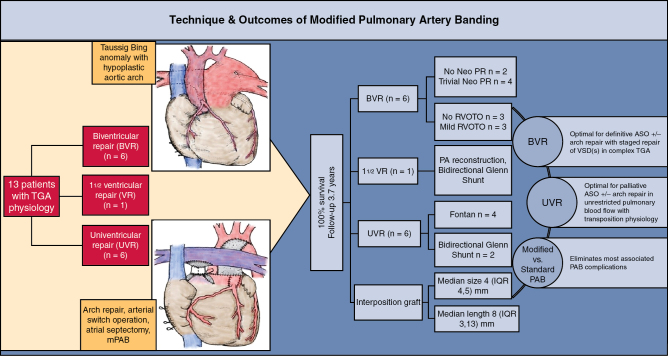
Methods, technique, results, and conclusions of modified pulmonary artery banding. *ASO*, Arterial switch operation; *BVR*, biventricular repair; *IQR*, interquartile range; *mPAB*, modified pulmonary artery banding; *Neo PR*, neopulmonary regurgitation; *PA*, pulmonary artery; *PAB*, pulmonary artery banding; *RV*, right ventricle; *RVOTO*, right ventricular outflow tract obstruction; *TGA*, transposition of the great arteries; *UVR*, univentricular repair; *VR*, ventricular repair; *VSD*, ventricular septal defect.

mPAB is a useful and reproducible technique in patients undergoing ASO who require concurrent restriction of antegrade pulmonary blood flow through the reconstructed neopulmonary root. This technique may reduce the degree of neopulmonary root distortion associated with standard PAB in this context and clearly eliminates the possibility of complications associated with distal band migration (Figure 3). Key to satisfactory outcome is tailoring interposition graft size and length to individual patient size and anatomy to achieve correct band “tightness” as determined by band gradient on subsequent echocardiography.

### Conflict of Interest Statement

The authors reported no conflicts of interest.

The *Journal* policy requires editors and reviewers to disclose conflicts of interest and to decline handling or reviewing manuscripts for which they may have a conflict of interest. The editors and reviewers of this article have no conflicts of interest.

## Supplementary Data

Video 1 Operative video demonstrating the technique of modified pulmonary artery banding (mPAB) in a neonate with d-transposition of the great arteries necessitating mPAB for straddling the mitral valve chordae across a large ventricular septal defect. Video available at: https://www.jtcvs.org/article/S2666-2507(21)00388-6/fulltext.
